# Supplementary material for: Uptake of Phosphate, Calcium, and Vitamin D by the Pregnant Uterus of Sheep in Late Gestation: Regulation by Chorionic Somatomammotropin Hormone
Source: Int J Mol Sci. 2022 Jul 14;23(14):7795. doi: 10.3390/ijms23147795 (PMC9320403; doi:10.3390/ijms23147795)
Supplement: Supplementary file 1 [file ijms-23-07795-s001.zip › Suppl Table S1.pdf]

Table S1. Effect of CSH RNAi on serum phosphate abundance.

	Control RNAi	CSH RNAi		Overall P Value
		Non-IUGR	IUGR	
**Uterine**				
Uterine Arterial Phosphate (nmol/µl)	1.861 ± 0.528	N/A	1.818 ± 0.554	>0.10
Uterine Venous Phosphate (nmol/µl)	1.924 ± 0.324	1.773 ± 0.190	1.677 ± 0.482	>0.10
Uterine Artery - Vein Phosphate Gradient (nmol/µl)	0.219 ± 0.463	N/A	-0.670 ± 0.686	>0.10
Uterine Phosphate Uptake (µmol/min)	2075 ± 715.4	N/A	1978 ± 418.9	>0.10
Uterine Phosphate Uptake per kg of Uterus (µmol/min)	1973 ± 655.4	N/A	3824 ± 858.4	>0.10
Uterine Phosphate Uptake per kg Fetus (µmol/min)	419.7 ± 125.2	N/A	642.3 ± 159.6	>0.10
Uterine Phosphate Uptake per kg Placenta (µmol/min)	3550 ± 895.1	N/A	5511 ± 1856	>0.10
**Umbilical**				
Umbilical Arterial Phosphate (nmol/µl)	2.754 ± 0.239	2.861 ± 0.250	2.419 ± 0.370	>0.10
Umbilical Venous Phosphate (nmol/µl)	2.443 ± 0.264	2.798 ± 0.367	2.120 ± 0.273	>0.10
Umbilical Vein – Artery Phosphate Gradient (nmol/µl)	-0.311 ± 0.271	-0.214 ± 0.410	-0.299 ± 0.219	>0.10
Umbilical Phosphate Uptake (µmol/min)	-260.0 ± 194.2	-261.9 ± 317.4	192.7 ± 96.37	>0.10
Umbilical Phosphate Uptake per kg of Uterus (µmol/min)	-301.7 ± 301.6	-467.5 ± 499.2	-346.4 ± 192.6	>0.10
Umbilical Phosphate Uptake per kg Fetus (µmol/min)	-64.05 ± 51.93	-65.76 ± 83.31	-53.59 ± 34.94	>0.10
Umbilical Phosphate Uptake per kg Placenta (µmol/min)	-576.0 ± 407.5	-618.2 ± 808.2	-410.8 ± 269.4	>0.10
**Uteroplacental**				
Uteroplacental Phosphate Utilization (µmol/min)	2198 ± 823.8	N/A	1506 ± 780.9	>0.10

Data are presented as means ± SEM. Different superscripts indicate statistical significance. (Control RNAi n = 4 - 10; CSH RNAi Non-IUGR n = 6; CSH RNAi IUGR n = 4). CSH, chorionic somatomammotropin; IUGR, intrauterine growth restriction; N/A, samples/results not available; RNAi, RNA interference.
