# Supplementary material for: Uptake of Phosphate, Calcium, and Vitamin D by the Pregnant Uterus of Sheep in Late Gestation: Regulation by Chorionic Somatomammotropin Hormone
Source: Int J Mol Sci. 2022 Jul 14;23(14):7795. doi: 10.3390/ijms23147795 (PMC9320403; doi:10.3390/ijms23147795)
Supplement: Supplementary file 1 [file ijms-23-07795-s001.zip › Suppl Table S2.pdf]

Table S2. Correlations between maternal, uterine, fetal, and placental weights and the abundance of phosphate and calcium in caruncles and cotyledons.

Tissue	Biometric Parameter	Mineral	r	P-Value
Caruncle	Maternal Weight	Calcium	0.121	0.133
Cotyledon	Maternal Weight	Calcium	0.07	0.273
Caruncle	Maternal Weight	Phosphate	0.001	0.884
Cotyledon	Maternal Weight	Phosphate	0.093	0.205
Caruncle	Uterine Weight	Calcium	0.035	0.432
Cotyledon	Uterine Weight	Calcium	0.017	0.597
Caruncle	Uterine Weight	Phosphate	0.028	0.493
Cotyledon	Uterine Weight	Phosphate	0.0004	0.934
Caruncle	Fetal Weight	Calcium	0.181	0.06
Cotyledon	Fetal Weight	Calcium	0.002	0.87
Caruncle	Fetal Weight	Phosphate	3.852e-005	0.98
Cotyledon	Fetal Weight	Phosphate	0.007	0.737
Caruncle	Placental Weight	Calcium	0.062	0.29
Cotyledon	Placental Weight	Calcium	0.163	0.09
Caruncle	Placental Weight	Phosphate	0.002	0.867
Cotyledon	Placental Weight	Phosphate	0.365	0.006
