# Supplementary material for: Uptake of Phosphate, Calcium, and Vitamin D by the Pregnant Uterus of Sheep in Late Gestation: Regulation by Chorionic Somatomammotropin Hormone
Source: Int J Mol Sci. 2022 Jul 14;23(14):7795. doi: 10.3390/ijms23147795 (PMC9320403; doi:10.3390/ijms23147795)
Supplement: Supplementary file 1 [file ijms-23-07795-s001.zip › Suppl Table S3.pdf]

Table S3. Correlations between maternal and uterine weights and calcium, phosphate, and 25(OH)D abundance, uptake, gradients, and uteroplacental utilization.

Biometric Parameter		Mineral/ Metabolite	r	P-Value
Maternal Weight	Uterine Artery (ng/mL)	calcium	0.103	0.168
Maternal Weight	Uterine Vein (ng/mL)	calcium	0.103	0.168
Maternal Weight	Umbilical Artery (ng/mL)	calcium	0.006	0.753
Maternal Weight	Umbilical Vein (ng/mL)	calcium	0.002	0.836
Maternal Weight	Uterine Uptake	calcium	0.093	0.191
Maternal Weight	Umbilical Uptake (ug/min)	calcium	0.047	0.360
Maternal Weight	Uterine Artery - Umbilical Artery Gradient	calcium	0.059	0.302
Maternal Weight	Uterine Artery - Uterine Vein Gradient	calcium	0.003	0.821
Maternal Weight	Umbilical Vein - Umbilical Artery Gradient	calcium	0.037	0.416
Maternal Weight	Uteroplacental Utilization (ug/min)	calcium	0.010	0.676
Maternal Weight	Uterine Artery (ng/mL)	phosphate	0.003	0.901
Maternal Weight	Uterine Vein (ng/mL)	phosphate	0.016	0.609
Maternal Weight	Umbilical Artery (ng/mL)	phosphate	0.002	0.858
Maternal Weight	Umbilical Vein (ng/mL)	phosphate	0.055	0.322
Maternal Weight	Uterine Uptake	phosphate	0.009	0.858
Maternal Weight	Umbilical Uptake (ug/min)	phosphate	0.030	0.476
Maternal Weight	Uterine Artery - Umbilical Artery Gradient	phosphate	0.009	0.820
Maternal Weight	Uterine Artery - Uterine Vein Gradient	phosphate	0.001	0.923
Maternal Weight	Umbilical Vein - Umbilical Artery Gradient	phosphate	0.072	0.253
Maternal Weight	Uteroplacental Utilization (ug/min)	phosphate	0.004	0.883
Maternal Weight	Uterine Artery (ng/mL)	25(OH)D	0.431	0.109
Maternal Weight	Uterine Vein (ng/mL)	25(OH)D	0.104	0.179
Maternal Weight	Umbilical Artery (ng/mL)	25(OH)D	0.008	0.709
Maternal Weight	Umbilical Vein (ng/mL)	25(OH)D	0.005	0.762
Maternal Weight	Uterine Uptake	25(OH)D	0.180	0.343
Maternal Weight	Umbilical Uptake (ug/min)	25(OH)D	0.051	0.338
Maternal Weight	Uterine Artery - Umbilical Artery Gradient	25(OH)D	0.371	0.146
Maternal Weight	Uterine Artery - Uterine Vein Gradient	25(OH)D	0.138	0.411
Maternal Weight	Umbilical Vein - Umbilical Artery Gradient	25(OH)D	0.002	0.858
Maternal Weight	Uteroplacental Utilization (ug/min)	25(OH)D	0.02	0.762

Uterine Weight	Uterine Artery (ng/mL)	calcium	0.002	0.843
Uterine Weight	Uterine Vein (ng/mL)	calcium	0.001	0.883
Uterine Weight	Umbilical Artery (ng/mL)	calcium	0.019	0.564
Uterine Weight	Umbilical Vein (ng/mL)	calcium	0.024	0.512
Uterine Weight	Uterine Uptake	calcium	0.033	0.442
Uterine Weight	Umbilical Uptake (ug/min)	calcium	0.174	0.067
Uterine Weight	Uterine Artery - Umbilical Artery Gradient	calcium	0.003	0.818
Uterine Weight	Uterine Artery - Uterine Vein Gradient	calcium	0.276	0.017
Uterine Weight	Umbilical Vein - Umbilical Artery Gradient	calcium	0.027	0.491
Uterine Weight	Uteroplacental Utilization (ug/min)	calcium	0.142	0.101
Uterine Weight	Uterine Artery (ng/mL)	phosphate	0.033	0.666
Uterine Weight	Uterine Vein (ng/mL)	phosphate	0.017	0.599
Uterine Weight	Umbilical Artery (ng/mL)	phosphate	0.004	0.799
Uterine Weight	Umbilical Vein (ng/mL)	phosphate	0.006	0.754
Uterine Weight	Uterine Uptake	phosphate	0.005	0.895
Uterine Weight	Umbilical Uptake (ug/min)	phosphate	0.017	0.599
Uterine Weight	Uterine Artery - Umbilical Artery Gradient	phosphate	0.024	0.712
Uterine Weight	Uterine Artery - Uterine Vein Gradient	phosphate	0.042	0.626
Uterine Weight	Umbilical Vein - Umbilical Artery Gradient	phosphate	0.016	0.599
Uterine Weight	Uteroplacental Utilization (ug/min)	phosphate	0.096	0.456
Uterine Weight	Uterine Artery (ng/mL)	25(OH)D	0.189	0.329
Uterine Weight	Uterine Vein (ng/mL)	25(OH)D	0.013	0.639
Uterine Weight	Umbilical Artery (ng/mL)	25(OH)D	0.002	0.862
Uterine Weight	Umbilical Vein (ng/mL)	25(OH)D	0.078	0.235
Uterine Weight	Uterine Uptake	25(OH)D	0.296	0.207
Uterine Weight	Umbilical Uptake (ug/min)	25(OH)D	0.032	0.451
Uterine Weight	Uterine Artery - Umbilical Artery Gradient	25(OH)D	0.003	0.904
Uterine Weight	Uterine Artery - Uterine Vein Gradient	25(OH)D	0.245	0.258
Uterine Weight	Umbilical Vein - Umbilical Artery Gradient	25(OH)D	0.121	0.145
Uterine Weight	Uteroplacental Utilization (ug/min)	25(OH)D	0.089	0.517
