# Supplementary material for: Uptake of Phosphate, Calcium, and Vitamin D by the Pregnant Uterus of Sheep in Late Gestation: Regulation by Chorionic Somatomammotropin Hormone
Source: Int J Mol Sci. 2022 Jul 14;23(14):7795. doi: 10.3390/ijms23147795 (PMC9320403; doi:10.3390/ijms23147795)
Supplement: Supplementary file 1 [file ijms-23-07795-s001.zip › Suppl Table S4.pdf]

Table S4. Correlations between fetal and placental weights and calcium, phosphate, and 25(OH)D abundance, uptake, gradients, and uteroplacental utilization.

Biometric Parameter	Variable	Mineral/ Metabolite	r	P-Value
Fetal Weight	Uterine Artery (ng/mL)	calcium	0.118	0.138
Fetal Weight	Uterine Vein (ng/mL)	calcium	0.068	0.266
Fetal Weight	Umbilical Artery (ng/mL)	calcium	0.044	0.377
Fetal Weight	Umbilical Vein (ng/mL)	calcium	0.044	0.375
Fetal Weight	Uterine Uptake	calcium	0.035	0.430
Fetal Weight	Umbilical Uptake (ug/min)	calcium	0.499	0.0005
Fetal Weight	Uterine Artery - Umbilical Artery Gradient	calcium	0.119	0.137
Fetal Weight	Uterine Artery - Uterine Vein Gradient	calcium	0.334	0.008
Fetal Weight	Umbilical Vein - Umbilical Artery Gradient	calcium	0.001	0.877
Fetal Weight	Uteroplacental Utilization (ug/min)	calcium	0.075	0.243
Fetal Weight	Uterine Artery (ng/mL)	phosphate	0.058	0.565
Fetal Weight	Uterine Vein (ng/mL)	phosphate	0.022	0.544
Fetal Weight	Umbilical Artery (ng/mL)	phosphate	0.089	0.214
Fetal Weight	Umbilical Vein (ng/mL)	phosphate	0.015	0.601
Fetal Weight	Uterine Uptake	phosphate	0.046	0.683
Fetal Weight	Umbilical Uptake (ug/min)	phosphate	0.068	0.280
Fetal Weight	Uterine Artery - Umbilical Artery Gradient	phosphate	0.0005	0.960
Fetal Weight	Uterine Artery - Uterine Vein Gradient	phosphate	0.133	0.375
Fetal Weight	Umbilical Vein - Umbilical Artery Gradient	phosphate	0.003	0.816
Fetal Weight	Uteroplacental Utilization (ug/min)	phosphate	0.483	0.056
Fetal Weight	Uterine Artery (ng/mL)	25(OH)D	0.436	0.108
Fetal Weight	Uterine Vein (ng/mL)	25(OH)D	0.294	0.016
Fetal Weight	Umbilical Artery (ng/mL)	25(OH)D	0.033	0.446
Fetal Weight	Umbilical Vein (ng/mL)	25(OH)D	0.035	0.430
Fetal Weight	Uterine Uptake	25(OH)D	0.090	0.514
Fetal Weight	Umbilical Uptake (ug/min)	25(OH)D	0.052	0.336
Fetal Weight	Uterine Artery - Umbilical Artery Gradient	25(OH)D	4.024e-005	0.989
Fetal Weight	Uterine Artery - Uterine Vein Gradient	25(OH)D	0.038	0.677
Fetal Weight	Umbilical Vein - Umbilical Artery Gradient	25(OH)D	0.004	0.799
Fetal Weight	Uteroplacental Utilization (ug/min)	25(OH)D	0.030	0.708

Placental Weight	Uterine Artery (ng/mL)	calcium	0.004	0.799
Placental Weight	Uterine Vein (ng/mL)	calcium	0.0001	0.966
Placental Weight	Umbilical Artery (ng/mL)	calcium	0.161	0.08
Placental Weight	Umbilical Vein (ng/mL)	calcium	0.181	0.06
Placental Weight	Uterine Uptake	calcium	0.033	0.444
Placental Weight	Umbilical Uptake (ug/min)	calcium	0.319	0.0095
Placental Weight	Uterine Artery - Umbilical Artery Gradient	calcium	0.098	0.179
Placental Weight	Uterine Artery - Uterine Vein Gradient	calcium	0.210	0.043
Placental Weight	Umbilical Vein - Umbilical Artery Gradient	calcium	0.065	0.278
Placental Weight	Uteroplacental Utilization (ug/min)	calcium	0.037	0.416
Placental Weight	Uterine Artery (ng/mL)	phosphate	0.045	0.615
Placental Weight	Uterine Vein (ng/mL)	phosphate	3.744e-005	0.980
Placental Weight	Umbilical Artery (ng/mL)	phosphate	0.029	0.488
Placental Weight	Umbilical Vein (ng/mL)	phosphate	0.007	0.733
Placental Weight	Uterine Uptake	phosphate	0.014	0.824
Placental Weight	Umbilical Uptake (ug/min)	phosphate	0.058	0.324
Placental Weight	Uterine Artery - Umbilical Artery Gradient	phosphate	0.004	0.888
Placental Weight	Uterine Artery - Uterine Vein Gradient	phosphate	0.007	0.537
Placental Weight	Umbilical Vein - Umbilical Artery Gradient	phosphate	0.034	0.434
Placental Weight	Uteroplacental Utilization (ug/min)	phosphate	0.312	0.150
Placental Weight	Uterine Artery (ng/mL)	25(OH)D	0.038	0.676
Placental Weight	Uterine Vein (ng/mL)	25(OH)D	0.072	0.267
Placental Weight	Umbilical Artery (ng/mL)	25(OH)D	0.020	0.549
Placental Weight	Umbilical Vein (ng/mL)	25(OH)D	0.027	0.491
Placental Weight	Uterine Uptake	25(OH)D	0.001	0.936
Placental Weight	Umbilical Uptake (ug/min)	25(OH)D	0.014	0.614
Placental Weight	Uterine Artery - Umbilical Artery Gradient	25(OH)D	0.067	0.576
Placental Weight	Uterine Artery - Uterine Vein Gradient	25(OH)D	0.0009	0.949
Placental Weight	Umbilical Vein - Umbilical Artery Gradient	25(OH)D	0.0001	0.961
Placental Weight	Uteroplacental Utilization (ug/min)	25(OH)D	0.184	0.337
