# Supplementary material for: Uptake of Phosphate, Calcium, and Vitamin D by the Pregnant Uterus of Sheep in Late Gestation: Regulation by Chorionic Somatomammotropin Hormone
Source: Int J Mol Sci. 2022 Jul 14;23(14):7795. doi: 10.3390/ijms23147795 (PMC9320403; doi:10.3390/ijms23147795)
Supplement: Supplementary file 1 [file ijms-23-07795-s001.zip › Suppl Table S5.pdf]

Table S5. Primer Sequences.

Gene Symbol	Gene Name	Accession Number		Primer Sequence (5'→ 3')	Tm (ºC)	Amplicon Size
ACTB	Beta-actin	NM_001009784.3	Fwd	CCACCGCAAATGCTTCTAGG	60	79
			Rev	CGTTTTCTGCGCAAGTTAGG		
ADAM10	A Disintegrin and metalloproteinase domain-containing protein 10	XM_004010565.4	Fwd	TGTGCCAGTTCTGATGGCAA	60	103
			Rev	TCCACTGCACAGACCCTGTA		
ADAM17	A Disintegrin and metalloproteinase domain-containing protein 17	XM_004005676.4	Fwd	ATGGCAAGTGTGAGAAGCGA	60	117
			Rev	GGACGGAACCGACGATGTTA		
ATP2B4	Plasma membrane calcium-transporting ATPase 4	XM_027976182.1	Fwd	TTGACAGCGGAAGGAGAGC	60	117
			Rev	CGTGGATCTTGCGGGAGTT		
CYP24	Cytochrome P450 Family 24 Subfamily A Member 1	XM_027976458.1	Fwd	AACGGTGGCTTCAGGACAAG	60	82
			Rev	CGACCCACGCACATTCTTTT		
FGFR1	Fibroblast growth factor receptor 1	XM_027962628.1	Fwd	ACAAGATGAAGAGCGGCACA	60	107
			Rev	GTCGGCTGACACTGTTACCT		
FGFR2	Fibroblast growth factor receptor 2	XM_027960356.1	Fwd	CCTGCGGAGACAGGTAACAG	60	137
			Rev	GCAGCTCATACTCGGAGACC		
GAPDH	Glyceraldehyde-3-phosphate dehydrogenase	NM_001190390.1	Fwd	GGGCAGCCCAGAACATCAT	60	112
			Rev	CCAGTGAGCTTCCCGTTCAG		
KL	Klotho	XM_004012279.4	Fwd	GCCTGCACATAGGGGACTTT	60	120
			Rev	CTCCAGCCACTGCGCTATAA		
PTHRP	Parathyroid hormone-related peptide	XM_004006756.3	Fwd	CCTAGTTCGCAAAGAAGCTGAC	60	71
			Rev	GCAGGGCTAACTCCTTCCTA		
SDHA			Fwd	CATCCACTACATGACGGAGCA	60	90

	Succinate dehydrogenase complex flavoprotein subunit A	XM_027980212.1	Rev	ATCTTGCCATCTTCAGTTCTGCTA		
SLC20A1	Solute carrier family 20 member 1	XM_004005910.4	Fwd	TGTGGTCTTCTATAAAAGGTGCG	60	121
			Rev	TCCATGGCGGAGAAGAGAAAG		
SLC20A2	Solute carrier family 20 member 2	XM_027962670.1	Fwd	GAGGAGGTACCGATGCGAG	60	119
			Rev	TTGCCTCATGACTGAGGTCG		
S100G	S100 calcium-binding protein G	XM_004021937.3	Fwd	GCAGCCAAAGAAGGTGATCC	60	73
			Rev	TGGGGAATTCCGTCTGAAGC		
S100A9	S100 calcium binding protein A9	XM_012181723.2	Fwd	AGGGTCAAAGGAAGCTTGACA	60	88
			Rev	TTTGTGACAATTGGTCCGCC		
STC1	Stanniocalcin 1	XM_004004182.4	Fwd	ACAAGATGGCGACCACCAAA	60	104
			Rev	AGTGACGCTCCTAAGGGACT		
STC2	Stanniocalcin 2	XM_027979996.1	Fwd	GGTGTTTTCCCCCGAGACAT	60	96
			Rev	GAAACAAAGCAGCGGCATGA		
TRPV6	Transient receptor potential cation channel subfamily V member 6	XM_004008129.4	Fwd	TTTGCGCGAGGATTCCAGAT	60	90
			Rev	CATCAGCCAGCAGAACCTCA		
TUB	Alpha-tubulin	XM_027967380.2	Fwd	GGTCTTCAAGGCTTCTTGGT	60	94
			Rev	CATAATCGACAGAGAGGCGT		
VDR	Vitamin D receptor	XM_027967403.1	Fwd	AGTTCGCAAGGATGAGGACG	60	84
			Rev	CAGGAGGACGACGAGTTTCC		
